# Fecal Iron Measurement in Studies of the Human Intestinal Microbiome

**DOI:** 10.1093/cdn/nzac143

**Published:** 2022-09-13

**Authors:** Afreen Z Khan, Sayema Badar, Karen M O'Callaghan, Stanley Zlotkin, Daniel E Roth

**Affiliations:** Department of Nutritional Sciences, Faculty of Medicine, University of Toronto, Toronto, Canada; Centre for Global Child Health and SickKids Research Institute, The Hospital for Sick Children, Toronto, Canada; Centre for Global Child Health and SickKids Research Institute, The Hospital for Sick Children, Toronto, Canada; Faculty of Health Sciences, Simon Fraser University, Burnaby, Canada; Centre for Global Child Health and SickKids Research Institute, The Hospital for Sick Children, Toronto, Canada; Department of Nutritional Sciences, Faculty of Medicine, University of Toronto, Toronto, Canada; Centre for Global Child Health and SickKids Research Institute, The Hospital for Sick Children, Toronto, Canada; Department of Paediatrics, The Hospital for Sick Children and University of Toronto, Toronto, Canada; Dalla Lana School of Public Health, University of Toronto, Toronto, Canada; Department of Nutritional Sciences, Faculty of Medicine, University of Toronto, Toronto, Canada; Centre for Global Child Health and SickKids Research Institute, The Hospital for Sick Children, Toronto, Canada; Department of Paediatrics, The Hospital for Sick Children and University of Toronto, Toronto, Canada; Dalla Lana School of Public Health, University of Toronto, Toronto, Canada

**Keywords:** iron, feces, microbiota, microbiome, iron deficiency, fecal iron, iron absorption, gastrointestinal iron

## Abstract

Iron is an essential micronutrient for humans and their intestinal microbiota. Host intestinal cells and iron-dependent bacteria compete for intraluminal iron, so the composition and functions of the gut microbiota may influence iron availability. Studies of the effects of the microbiota or probiotic interventions on host iron absorption may be particularly relevant to settings with high burdens of iron deficiency and gastrointestinal infections, since inflammation reduces iron bioavailability and unabsorbed intraluminal iron may modify the composition of the microbiota. The quantification of stool iron content may serve as an indicator of the amount of intraluminal iron to which the intestinal microbiota is exposed, which is particularly relevant for studies of the effect of iron on the intestinal microbiome, where fecal samples collected for purposes of microbiome characterization can be leveraged for stool iron analysis. However, few studies are available to guide researchers in the selection and implementation of stool iron assays, particularly because cross-comparison of available methods is limited in literature. This review aims to describe the available stool iron quantification methods and highlight their potential application in studies of iron–microbiome relationships, with a focus on pediatric research. MS-based methods offer high sensitivity and precision, but the need for expensive equipment and the high per-sample and maintenance costs may limit their widespread use. Conversely, colorimetric assays offer lower cost, ease of use, and rapid turnaround times but have thus far been optimized primarily for blood-derived matrices rather than stool. Further research efforts are needed to validate and standardize methods for stool iron assessment and to determine if the incorporation of such analyses in human microbiome studies *1*) yields insights into the interactions between intestinal microbiota and iron and *2*) contributes to the development of interventions that mitigate iron deficiency and promote a healthy microbiome.

## Introduction

Iron is an essential micronutrient for most living organisms because of its role as a cofactor for enzymatic activity and electron transfer reactions (1, 2). In humans, iron is a component of hemoglobin (Hb) and myoglobin, a cofactor in DNA synthesis and repair, and is required in iron–sulphur cluster proteins involved in cellular respiration (3, 4). Infants have relatively high iron requirements as compared with older children and adults (5, 6) to support their relatively rapid blood volume expansion and brain development (1, 3). Iron is also required by most bacteria for growth and survival and is known to regulate virulence genes among pathogenic strains (7). Lactobacilli are unique in that they rely on manganese instead of iron for survival (8, 9); however, lactobacilli may enhance host iron absorption by secreting lactic acid, which lowers the intestinal pH and facilitates the reduction of ferric iron (Fe^3+^) to its more bioavailable ferrous (Fe^2+^) form (10, 11). Conversely, iron-scavenging bacteria chelate iron in the Fe^3+^ and Fe^2+^ forms through separate uptake mechanisms and leverage the availability of insoluble (unabsorbed) Fe^3+^ to promote their colonization of the intestinal microbiome (12). Microbial diversity can therefore influence iron bioavailability, whereas unabsorbed (intraluminal) iron can affect the composition and activity of the intestinal microbiota (13). Given the potential impact of unabsorbed intraluminal iron on the microbiome, quantification of fecal iron may be useful to further understand the iron–microbiome relationship and its association with health outcomes, including iron deficiency (ID).

The quantity of fecal iron is modified by various dietary and gastrointestinal factors that affect iron bioavailability and enterocyte function (14–16), including the composition and activity of intestinal microbiota. However, the extent to which the quantification of iron in stool can enhance our understanding of gastrointestinal iron utilization is underexplored. The routine collection of stool samples in microbiome research studies provides an opportunity to examine the iron–microbiota interactions that may modify intestinal iron loss. Laboratory methods to examine fecal iron content were first developed in the 1930s (17) and have been used to quantitatively measure iron balance. Yet, according to a Medline database search that was initially completed in April 2021 and updated until August 2022, we identified very few studies that inform the selection of appropriate analytic methods (**Table 1**). In this review, we first consider the rationale for measuring stool iron content in human microbiome studies by reviewing *1*) relevant aspects of iron absorption and metabolism, *2*) determinants of fecal iron content, *3*) interactions between iron and intestinal microbiota, and *4*) evidence from trials regarding the effects of iron supplementation on the microbiota and the effects of probiotic or prebiotic interventions on iron absorption. Then, we compare the available assays that have been used for fecal iron assessment, with a particular focus on their application in pediatric research.

**TABLE 1:** Stool iron content measurements in infants or children reported in the literature^1^

Author Year	Location	Participants, *n*	Age Range	Instrument/Technique	Feeding Type or Intervention	Detection of Stool Iron^2^
MacDougall 1970 (153)	USA	466	5 d–14 y	Colorimetry: qualitative iron staining method that stains positive samples blue (chromogen: ferrous ferricyanide)	No iron supplementation	3% of samples had detectable iron
					Iron-fortified formula (12 mg Fe)	87% of samples had detectable iron
					Oral iron supplementation (15–66 mg Fe)	89% of samples had detectable iron
Pizarro 1987 (58)	Chile	310	3–15 mo	Colorimetry: ashing method to free chelated iron, followed by colorimetric measurement via addition of chromogen and detecting absorbance	Iron-fortified formula (15 mg ferrous sulphate/L)	27.0 ± 9.6 mg Fe/100 g stool
					Non–iron-fortified formula	8.9 ± 3.7 mg Fe/100 g stool^3^
Fairweather-Tait 1987 (55)	England	29	7–10 d	AAS: ashing method to free chelated iron, followed by AAS via PU9000 AAS (Pye Unicam)	Iron-fortified formula (40 µg Fe/100 mL), followed by Fe^58^ FeCl_3_	7.24 ± 4.26 mg Fe/100 g dry weight of stool
					Iron-fortified formula (40 µg Fe/100 mL), followed by Fe^58^ lactoferrin	8.82 ± 2.91 mg Fe/100 g dry weight of stool
					Iron-fortified formula (86 µg Fe/100 mL) followed by Fe^58^ FeCl_3_	10.86 ± 4.84 mg Fe/100 g dry weight of stool
					Iron-fortified formula (86 µg Fe/100 mL) followed by Fe^58^ lactoferrin	10.19 ± 1.42 mg Fe/100 g dry weight of stool
Qasem 2017 (57)	Canada	82	4–6 mo	Colorimetry: digestion by acid mix (Fisher Scientific Inc) to extract fecal iron, followed by spectrophotometric measurement with Feren-B kit (Bioanalytic)	EBF, before CF (cereal)	3.9 ± 0.37 g Fe/stool
					EBF followed by CF (cereal containing 8.8 mg Fe/28 g cereal)	5.6 ± 0.38 g Fe/stool
					EBF, before CF (cereal + fruit)	3.9 ± 0.32 g Fe/stool
					EBF followed by CF (cereal + fruit containing 8.8 mg Fe/28 g cereal)	4.7 ± 0.34 g Fe/stool
					EBF, before CF (meat)	2.9 ± 0.25 g Fe/stool
					EBF followed by CF (meat containing 1.3 mg/100 mL jar)	3.7 ± 0.25 g Fe/stool^3^^,^^4^
Kalipatnapu 2017 (69)	India	40	2–5 y	Colorimetry: freeze-drying, followed by ashing method to free chelated iron and addition of chromogen (Fisher Diagnostics) to detect absorbance	Rural regular diet (2.3 mg Fe/d)	365.6 (286.0–555.7) mg Fe/100 g wet weight of stool
					Urban regular diet (2.6 mg Fe/d)	11.49 (5.0–32.7) mg Fe/100 g wet weight of stool^5^
Paalanne 2018 (128)	Finland	18	21.8 ± 30.6 mo, controls	ICP-OES: freeze-drying method, followed by microwave digestion (CEM MARS 5X) and then spectrometric reading via ICP-OES	Breastfed for 3–4 mo	40.3 ± 36.7 mg Fe/100 g dry weight of stool (no febrile UTI)
			20.3 ± 27.2 mo, cases			40.6 ± 30.2 mg Fe/100 g dry weight of stool (febrile UTI)

1 Values are presented as mean ± SD unless noted otherwise. AAS, atomic absorption spectrophotometry; CF, complementary feeding; EBF, exclusively breastfed; ICP-OES, inductively coupled plasma optical emission spectrometry; UTI, urinary tract infection.

2 Units for stool iron concentration have been converted to mg Fe/100 g stool, where applicable, for comparability of results. Wet and dry weight may not be comparable due to the variation in water content of stool samples.

3 Mean ± SE.

4 Measurements were based on a starting stool weight of 300–400 mg; the denominator for the final reported concentration is unclear.

5 Difference between rural and semiurban areas does not appear plausible, but we were unable to receive further confirmation from the author; results are reported as median (IQR).

## Current Status of Knowledge

### Early-life determinants of iron status

Neonatal iron stores are acquired by transplacental transfer of maternal iron to the fetus, predominantly in the third trimester (18–20). As gestational age and fetal growth directly influence iron accretion (21, 22), prematurity and a low birth weight are primary risk factors for neonatal ID, whereas delayed umbilical cord clamping has been shown to substantially increase iron stores at birth (23–25). Breastmilk iron is highly bioavailable (∼50%) but has a low iron content (∼0.47 mg/L) (26, 27). When maternal iron stores are adequate, the combination of iron accumulated during the last trimester and bioavailable iron from breastmilk is considered sufficient to meet the iron requirements of the full-term infant for the first 6 mo of life. Additional dietary sources of iron are required thereafter; hence, exclusive breastfeeding beyond 6 mo and the late introduction of iron-rich complementary foods are key risk factors for ID in infancy (28, 29).

### Iron homeostasis and the determinants of stool iron content

Nonheme dietary iron is in the Fe^3+^ state but reduced to Fe^2+^ by the low pH of the stomach before it reaches the duodenum (30). Reduction of Fe^3+^ is required for entry to enterocytes, but the high pH of the distal intestine can oxidize iron back to its Fe^3+^ state (31). In the duodenum, nonheme Fe^3+^ is reduced to Fe^2+^ by an apical membrane–associated Fe^3+^ reductase, duodenal cytochrome B (DCYTB) (32). Reduced iron enters the enterocyte via the channel protein divalent metal transporter 1 (DMT1), located on the surface of the intestinal cell membrane. DMT1 is considered the major route for nonheme iron absorption into the enterocyte. Intracellular Fe^2+^ is stored as ferritin, the main intracellular iron storage protein, or it travels to the basolateral layer of the enterocyte to be released into the bloodstream via the exporter protein ferroportin. The export of Fe^2+^ is preceded by its oxidation back to Fe^3+^ by a ferroxidase, hephaestin; the extracellular iron is then bound to transferrin for delivery to target tissues (33). Heme iron, though, is more bioavailable as it is not influenced by gastrointestinal tract pH or by inhibitors or promoters that would otherwise hinder nonheme iron absorption (34). However, the mechanisms of heme iron absorption are yet to be fully understood.

Although the enterocyte brush border transporter HCP1 (heme carrier protein 1) was earlier shown to mediate the transport of heme iron into the intestinal cell, this protein was later found to have a higher affinity for folate and is mainly involved in folate transport (35, 36). More recently, HRG1 (heme-responsive gene 1) has been proposed as another putative heme transporter (37, 38). Within the enterocyte, heme undergoes degradation by heme oxygenase, and the iron that is released from the protoporphyrin ring enters the nonheme iron pool (39).

Nonheme iron absorption is regulated by endocrine control of ferroportin activity (40, 41). At adequate or high-circulating iron concentrations, the hormone hepcidin is released from the liver and binds directly to ferroportin at the basolateral membrane, thereby blocking iron transfer into the circulation (42). Although iron absorption predominantly takes place in the duodenum, human and animal studies have reported expression of DMT1 and ferroportin in the distal intestine and colon, acting as a compensatory mechanism to upregulate iron absorption under low iron conditions (43–47). Isotope studies involving an orally administered dose of labeled iron revealed that iron absorption occurs in 2 sequential steps: a rapid phase of absorption that occurs within the first 2 h in the small intestine (mainly duodenum and jejunum), followed by a slower phase of absorption (approximately 24 h) in the distal small bowel and colon (48–51). However, evidence of colonic iron absorption in infants is lacking.

There is no regulated physiologic process for excretion of absorbed iron; therefore, iron balance is primarily regulated at the level of absorption under the actions of hepcidin, as described earlier. Endogenous losses through sweat, urine, and enteric desquamation (shedding of intestinal cells) contribute to about 1–2 mg iron loss/d in adults (52, 53).

When the demand for iron in target tissues is low, iron absorbed into intestinal epithelial cells accumulates as ferritin for storage and hence may be lost when the enterocyte is shed in the stool (54). Although studies showing a direct comparison of adult and infant stool iron are not available, existing data corroborate the expectation of higher stool iron concentrations in adults relative to infants: Fairweather-Tait et al. (55) reported an average of ∼7 mg Fe/100 g dry weight of stool in infants aged 7–10 d who were fed iron-fortified formula containing 40 µg Fe/100 mL, whereas Lund et al. (56) reported that average stool iron concentrations among adult men and women were 97 and 99 mg Fe/100 g dry weight of stool, respectively, following 2-wk iron supplementation with 100 mg Fe^2+^ sulphate/d. The amount of iron lost among infants depends on the amount and bioavailability of dietary iron such that a diet based exclusively on breastmilk yields very different concentrations of stool iron when compared with diets that include iron-fortified food or formulas, as demonstrated by Qasem et al. (57) and Pizarro et al. (58). Under normal physiologic conditions, a large proportion of intraluminal iron may remain unabsorbed, as the fractional absorption of nonheme iron is typically <20% (59, 60), which explains why unabsorbed iron is commonly detected in stool under healthy conditions. The bioavailability of nonheme iron depends on the food matrix; for example, absorption is promoted by ascorbic acid (60–62) and inhibited by phytates (60, 63). For supplemental iron, the form of the Fe^2+^ salt also influences fractional absorption (14, 64, 65). It is expected that dietary factors that influence iron uptake or bioavailability (i.e., inhibitors or promoters of iron absorption) and other characteristics of the gastrointestinal environment would modify the amount of iron detectable in stool, but the importance of such factors remains to be demonstrated empirically (**Table 2**).

**TABLE 2:** Factors that may affect stool iron content

	Factors Likely to	
	Increase Stool Iron by Decreasing Iron Absorption	Decrease Stool Iron by Increasing Iron Absorption
Dietary factors	• Infant formula (154)	• Breastmilk (21, 157)
	• Bovine milk proteins (155)	• Ascorbic acid (60)
	• Egg proteins (155)	• Citric acid (155)
	• Phytates (60)	• Lactic acid (155)
	• Polyphenols (60)	• Meat (155)
	• Oxalates (155)	
	• Calcium (60, 156)	
Intestinal microbiota	• Iron-dependent bacteria (*Escherichia coli, Clostridium* spp.) (13)	• Iron-independent bacteria (*Lactobacilli*) (69)
Other factors	• Inflammation (158)	• Hemochromatosis (159)
	• Intestinal sloughing (33)	
	• Intestinal bleeding (82, 83)	

Infection or inflammation can dysregulate iron homeostasis, in part by promoting hepcidin expression resulting in fecal iron loss. Hepcidin is an acute-phase reactant for which expression is induced by proinflammatory cytokines (e.g., IL-6) (66), which in turn suppresses iron absorption from the gastrointestinal lumen. Inflammation may also hinder iron absorption through hepcidin-independent pathways; in vitro evidence suggests that tumor necrosis factor α downregulates the expression of DMT1 on the intestinal epithelial cell, subsequently reducing iron absorption (67, 68) and potentially increasing the iron content of feces. As discussed in detail later, fractional iron absorption is influenced by the intestinal microflora; specifically, fecal loss of iron may be increased in the presence of iron-dependent bacteria (14–16) and conversely decreased by iron-independent bacteria, including lactobacilli (69).

Although most iron in stool represents unabsorbed ingested iron, variable quantities of iron may be lost in the form of Hb due to microscopic or macroscopic bleeding of the intestinal mucosa. Some parasitic infections may directly injure the intestinal mucosa, resulting in intestinal bleeding that contributes further to fecal iron loss (70, 71). Hookworm infection, commonly present in low-income settings, is a widely known cause of intestinal blood loss and as a result increases the risk of iron deficiency anemia (IDA) (70, 72). *Helicobacter pylori* infection specifically poses a risk of upper gastrointestinal bleeding through the development or aggravation of bleeding peptic ulcers (73), thereby contributing to Hb in stool, and has been reported to impair iron uptake by the host (74). However, loss of blood via stool may occur in the absence of a gastrointestinal infection, and intestinal bleeding, even in microscopic amounts, may contribute to the risk of ID (75–77). In early infancy, microscopic bleeding is mainly related to feeding patterns that promote exposure to bovine proteins, whereby gastrointestinal blood loss has primarily been observed in infants who switched from breastmilk to cow's milk (78–81). Bovine proteins in cow's milk include caseins, which bind to Fe^2+^ in the gastrointestinal tract, thereby inhibiting nonheme iron absorption (82). By using qualitative fecal occult blood tests, the presence of blood in infant stool has been reported among breastfed infants in Brazil, which the authors attributed to infant exposure to antigens from cow's milk consumed by the mother (83).

### Interactions between intestinal iron and the gut microbiota

Iron is an essential element for a variety of bacteria for which it serves as a cofactor in metabolic pathways and electron transport chain reactions (12, 84). The ability to utilize Fe^3+^ and Fe^2+^ contributes to the virulence of most pathogenic bacteria (12, 85, 86). Many bacterial species rely on extracellular iron-chelating proteins known as siderophores for direct uptake of Fe^3+^ (12); these proteins bind to iron in the surrounding microenvironment, and the resulting Fe^3+^–siderophore complex is engulfed by specific bacterial receptors (31). Many lactobacilli are unique in that they can survive without iron (8), instead using manganese as a cofactor in enzymatic reactions (87). *Lactiplantibacillus plantarum* was the first species to be identified as iron independent (8), whereas some bifidobacteria were later found capable of limiting access to iron by pathogens (31, 88). These commensals may be consumed by humans as probiotics, and some studies have suggested that they may improve nutrient absorption, including iron (89).

The effects of prebiotics (90, 91) and probiotics (10, 11, 14, 92–95) on iron absorption have been investigated in several studies. Data from rodent models suggest that addition of a probiotic containing *Bifidobacterium bifidum* and *B. longum*, a prebiotic (galacto-oligosaccharides), or a synbiotic (bifidobacteria and galacto-oligosachcarides) to infant formula can improve iron absorption in weanling rats when compared with a control diet, as measured via iron intake and fecal iron quantification (90). Increased iron bioavailability and duodenal iron content were also observed with multispecies probiotic supplementation in 10-wk-old Wistar rats (95). However, there is limited evidence from randomized trials examining the direct effects of probiotics on iron-related biomarkers in humans. Administration of *L. plantarum* 299v has been shown to improve iron absorption from fortified beverages (10) and meals (93) among adult females, reported as an increase in the percentage of iron absorption through the use of a double-isotope technique. A higher mean cell Hb concentration was found among preschool children following consumption of a milk beverage fortified with *Lactobacillus acidophilus*, although other parameters, including serum ferritin, were unaffected (96). The hypothesized increase in iron bioavailability that follows probiotic administration is believed to be a consequence of elevated acid production within the intestine; in vitro work by Gonzalez et al. (11) using IEC6 epithelial rat small intestinal cells demonstrated excretion of p-hydroxyphenyllactic acid by *Limosilactobacillus fermentum*, which reduces Fe^3+^ , thereby mimicking the activity of DCYTB in the gastrointestinal tract. Similarly, in vitro analysis by Sandberg et al. (94) showed an increased expression of DCYTB in human intestinal cell lines when incubated with a digested iron-fortified oat drink containing encapsulated lyophilized *L. plantarum* 299v. Dietary iron enters the intestine from the acidic environment of the stomach, but intestinal absorption requires Fe^3+^ to remain solubilized; therefore, promoting the reduction of Fe^3+^ to Fe^2+^ by lactic acid–secreting bacteria may contribute to iron uptake by the host (14, 97). Bering et al. (98) suggested that noncolonizing lactobacilli may release lactic acid during their transit through the intestinal tract. As previously mentioned, at least some iron absorption may occur in the colon (∼14% that of duodenal absorption) (16, 43, 46), possibly facilitated by a reduction in the pH and an increase in the crypt depth of the proximal colon following probiotic and/or synbiotic administration (90).

### Effect of supplemental iron on the infant gut microbiota

Iron–microbiota interactions are potentially important considerations in the assessment of the safety and efficacy of intervention programs to address ID across high-risk settings (13). Where the prevalence of anemia exceeds 40%, the WHO recommends provision of daily infant iron supplements for a consecutive 3-mo period (99). Supplementation containing 10–12.5 mg of elemental iron is advised from 6 to 23 mo of age, following the recommended period of exclusive breastfeeding (99). Where the prevalence of anemia is >20% but <40%, the recommendation is to practice home fortification with iron-containing micronutrient powders for 90 d every 6 mo (100).

Although oral iron administration is the most common strategy for addressing ID and IDA and has shown benefits in terms of IDA reduction and improvement in iron status (101–104), the design of an optimal strategy continues to be challenging in regions with high burdens of malaria and other infectious diseases. In some settings, universal iron supplementation has been observed to increase the risk of diarrhea, respiratory infections, and malaria (105). Diarrhea, which is the most common of these adverse effects (106–111), has been attributed in some studies to the alteration of colonic microbiota following iron supplementation and/or fortification (13, 31, 105, 112). Explanations for the increased risk of diarrhea are based on the understanding that excess intraluminal iron promotes redox stress (113, 114), in turn reducing the membrane permeability such that pathogenic bacteria may more readily translocate across the epithelial barrier (113, 114).

Coadministration of a probiotic and/or prebiotic with iron supplements may counterbalance the gastrointestinal effects of excess iron (115, 116). Probiotic bacteria such as specific strains of *L. plantarum, Escherichia coli Nissle*, and *Bifidobacterium infantis* (117) may contribute to the protection of the gastrointestinal environment (118), including relative inhibition of colonization by pathogenic (generally iron-utilizing) bacteria (119). Lin et al. (115) demonstrated an increase in fecal iron loss, increased liver load of *Salmonella*, and impairment of intestinal lining following iron fortification among weaning mice, whereas supplementation with the probiotic *L. acidophilus* or prebiotic inulin was able to mitigate these negative effects. The effects of probiotics may be enhanced when combined with prebiotics that promote the proliferation and activity of specific species of probiotic bacteria, such as bifidobacteria and lactobacilli (120, 121). Paganini et al. (64) reported increased iron absorption and enhanced growth and survival of bifidobacteria and lactobacilli when Kenyan infants were fed maize porridge fortified with a micronutrient powder containing a prebiotic (7.5 g of galacto-oligosaccharides) (64, 116). A recent review also highlighted that prebiotics, particularly galacto-oligosaccharides and fructo-oligosaccharides, have an enhancing effect on iron absorption, although several other factors can modify this association, such as dose of the prebiotic, age of population, underlying infection, iron status, and the form of iron consumed through diet or supplementation (91).

### Incorporation of fecal iron measurement in human microbiome studies

Serum or plasma ferritin is the most commonly used indicator of iron status, as it correlates strongly with intracellular iron stores (122). However, there are numerous other direct and indirect measures of iron status (e.g., serum iron, transferrin saturation, soluble transferrin receptor). Since there is no single biomarker that reflects all aspects of iron homeostasis, a panel of iron-related biomarkers is often used. Incorporation of stool iron measures in such a panel may provide specific insights into iron–microbiome interactions, particularly in the context of a randomized controlled trial of an iron and/or probiotic intervention. For example, if iron absorption increases as a direct effect of an intervention, a relative decrease in average iron losses can be expected in the intervention group as compared with the control group. Fecal iron analyses may also enable between-group comparisons in the context of an observational epidemiologic study, where factors that might influence iron intake and bioavailability could be included as covariates in a multivariable model. In addition, in settings where low adherence to interventions is of concern, fecal iron may be used as an indicator of adherence. Stool samples collected for metagenomic or metabolomic analyses can be leveraged for the analysis of fecal iron. Particularly for infants, fecal iron measurement has the advantage of relying on noninvasive specimen collection, in contrast to blood sampling, which is challenging in pediatric studies and for which repeated longitudinal sampling is unlikely to be feasible or acceptable. However, there are challenges associated with collection and processing of stools that are relevant to iron content analysis. Due to the reduction in stool mass during freeze-drying, sample requirements are greater than those required for microbiome analyses; for example, our experience has been that at least 2–3 g of stool are required to yield the minimum 0.2 g of stool needed for analysis by atomic absorption spectrophotometry (AAS). Sample transfers before and after freeze-drying unavoidably lead to a minor amount of sample loss. This may compromise the precision with which iron content can be expressed in terms of wet stool weight, although this can be overcome by normalizing measures to dry weight of stool, which also addresses between-sample variation due to stool water content. Other sources of within-sample and within-subject variance (e.g., diurnal variation) in stool iron content have not been previously explored but are likely important and would be expected to affect the participant sample sizes required to detect between-group differences in trials or observational studies. Another consideration is that, like most other biomarkers of iron status, fecal iron content is expected to be influenced by the acute-phase response (i.e., inflammation leading to increased hepcidin would be expected to decrease iron absorption and therefore increase stool losses).

The potential utility of fecal iron measurements to study iron–gut microbiota interactions was demonstrated by Kalipatnapu et al. (69) in 2017, who found an inverse association between fecal iron concentrations and relative abundance of lactobacilli among a cohort of 40 children aged 2 to 5 y in a rural and semiurban area of South India. Although the inverse association might be explained by the beneficial effects of lactobacilli on iron bioavailability and uptake, as proposed previously (10, 11, 92–94, 96), it is plausible that excess unabsorbed iron promotes the growth of iron-dependent bacteria, thereby reducing the relative abundance of beneficial commensals such as lactobacilli (123, 124). The association between beneficial commensals, including lactobacilli and bifidobacteria, and iron absorption has been examined in children and adults by using either stable isotopes to directly measure iron absorption (10, 93, 98, 125) or serum biomarkers such as ferritin, serum transferrin, or zinc protoporphyrin as a measure of iron status (126, 127). Although rarely reported as the primary study outcome, stool iron has been measured in several other studies as a secondary outcome to facilitate interpretation of intervention effects on iron bioavailability (55, 57), oxidative stress (57), or changes in microbiota composition (57, 128) in pediatric populations.

### Stable isotope techniques for measurement of iron absorption

The historical use of orally administered radioisotope tracers and more recent use of stable isotopes (mainly Fe^57^ and Fe^58^) have contributed substantially to the understanding of iron absorption in humans (129, 130). Application of stable isotope methods for detection of iron loss shares the challenges of many micronutrient tracers, including the need for large quantities of tracer doses to achieve adequate enrichment ratios (131), high cost and participant burden, as well as the technical expertise and resources required to use and maintain equipment such as mass spectrometers. The assessment of iron absorption requires intake of a labeled iron compound or food, followed by measurement of iron incorporation in erythrocytes (132) or fecal excretion of the labeled isotope over several days (typically 7–10 d postdosing) to allow complete recovery of the unabsorbed isotope from the gastrointestinal tract (133, 134). As such, this process is time-consuming and can be practically challenging in large-scale studies (135). Fecal iron measurements do not reflect fractional iron absorption at the individual level, but in research studies involving between-group comparisons, use of fecal iron may be a relatively cost-effective approach to enable comparisons of relative differences in iron absorption, where the direct measurement of iron absorption with stable isotopes is considered un-feasible.

### Colorimetric and spectrophotometric methods for measurement of fecal iron

Various techniques have been successfully employed for the direct quantification of iron in stool (**Table 3**). However, cross-comparison of methods has been little explored to date (17). Quantification of fecal iron retention was first determined through colorimetric- and titrimetric-based methods, but such methods were later criticized due to risk of interference by other elements (e.g., calcium, phosphorous), leading to underestimation of the true iron content (17). Most of the measurement techniques employed today are based on the method developed by Pizarro et al. (58) >3 decades ago, who proposed a rapid, simple, and inexpensive colorimetric technique suitable for field trials. Their method requires use of a muffle furnace to ash the stool sample for release of chelated iron. The ash is then dissolved into hydrochloric acid, followed by addition of a buffered chromogen, bathophenanthroline disulphonate disodium salt, and absorbance is then measured at 540 nm (58). The ashing technique met the standards set by the AOAC (136), and the selected chromogen (bathophenanthroline) showed increased sensitivity to iron when compared with potassium thiocyanate, which was previously used (58, 137).

**TABLE 3:** Characteristics of commonly used methods for detection of iron in stool samples^1^

Type of Assessment	Description	Attributes
AAS (146, 147)	Flame or graphite furnace AAS enables quantitative or qualitative determination of element concentrations by measuring the amount of energy absorbed by the sample from specific wavelengths of light	• High throughput
		• Measures only 1 element at a time
		• Graphite furnace AAS is slower than flame AAS
		• Large sample volume required vs. ICP-OES and ICP-MS
ICP-OES (146, 147)	Samples are ionized, inducing atoms to move to an excited state. As the atoms return to a lower energy state, they release radiation, the wavelengths of which are specific to each element. The intensity of each wavelength reflects the number of atoms of each element making the transition and can therefore be used to determine the concentration of that element in the sample	• High throughput
		• High instrumentation cost vs. AAS
		• Can measure multiple elements simultaneously
		• Higher possibility of spectral interference vs. AAS and ICP-MS
ICP-MS (146, 147)	Samples are ionized, and the ions themselves are detected when passed through a mass spectrometer, as the ions are sorted by mass. This technique therefore identifies type and quantity of specific molecules based on the mass:charge ratio	• High throughput
		• High instrumentation cost vs. AAS and ICP-OES
		• Can measure multiple elements simultaneously
		• Measurement range is larger than AAS and ICP-OES
		• Detection limit better than AAS and ICP-OES
Colorimetric assay (69)	Ferrous or ferric ions in samples are detected by their reaction with a chromogen. The acidic buffer allows any ferric iron to dissociate in its ferrous form, which can therefore react with Ferene chromogen to produce a stable blue-colored complex and an absorbance that is proportional to the concentration of iron in the sample	• No requirement for dedicated laboratory or expensive equipment
		• Low cost vs. automated instrumentation
		• Kits mainly designed for blood-derived fluids

1 AAS, atomic absorption spectrophotometry; ICP-MS, inductively coupled plasma mass spectrometry; ICP-OES, inductively coupled plasma optical emission spectrometry.

Although commercial colorimetric assay kits for total iron quantification are now widely available, these methods have been mainly designed for use with blood-derived fluids. There are limited reports of the use of such assays for fecal iron quantification, which necessitates additional presample processing as described earlier (58). These assays involve use of an acidic buffer to allow dissociation of Fe^3+^from its protein complex, followed by reduction into Fe^2+^ (138). Chromogens can then bind to the Fe^2+^ , resulting in a detectable color change (138). Although thiocyanate and bathophenanthroline were traditionally used as chromogens, ferrozine and ferene are now more commonly used for the detection of serum and stool iron, given their lower cost and comparable performance characteristics (139, 140). Direct comparison of ferrozine and ferene show linear associations with bathophenanthroline over a range of iron concentrations in serum (4.5–143.1 µmol/L; *r *= 1.000; *P *< 0.0001 for both) (140). Ferene is now considered preferential given its greater sensitivity and has shown negligible interference by other substances, such as copper, Hb, and bilirubin (141). As a result, most commercial kits available today include ferene as the chromogen of choice for determination of iron in biological fluids and fecal matter (57, 69, 142).

AAS, inductively coupled plasma mass spectrometry (ICP-MS), and, more recently, inductively coupled plasma optical emission spectrometry (ICP-OES) may also be used to measure fecal iron concentrations (130, 143, 144). Following sample processing as outlined by Pizarro et al. (58), AAS, ICP-MS, and ICP-OES can be employed for direct measurements of total iron concentration in feces (56, 128, 145), although the capital and running costs for flame and furnace AAS are relatively low when compared with ICP-OES and ICP-MS. The rapid processing time of flame AAS (<15 s/sample) makes it efficient for single-element analysis, whereas ICP-MS is particularly suited to multielement analysis given its capacity to analyze >70 elements in a short time frame (∼1–5 min) (146, 147). In earlier studies, freeze-dried and ashed fecal samples were analyzed by neutron activation analysis (148, 149), but this method has been sparingly mentioned in published literature, likely due to the limited access to a source of neutrons, such as nuclear reactors, the production of a radioactive sample during the process, and the need for highly skilled technicians (150).

Abbasi et al. (151) recently derived a method for the quantification of total iron as well as labile iron, also known as redox-active iron, using a bench-top colorimetric assay in a range of specimens, including feces, and reported sensitivity comparable to ICP-MS. The authors emphasized that previous colorimetric methods were mostly compatible with blood-derived samples and could detect total iron only, whereas their proposed unified ferene assay was capable of quantifying labile and total iron simultaneously. In brief, total iron measurement required an initial drying process at 100–200 °C, followed by acid digestion with concentrated nitric acid. The drying and reconstitution in acid were repeated; the samples then were mixed with a working solution containing a ferene chromogen; and values were detected by absorbance at 595 nm. The unified ferene assay was validated against ICP-MS across a variety of specimens (plasma, urine, organ samples, and feces), demonstrating high precision for total iron analysis (151).

Despite substantial progress in the development of fecal iron assessment methods in the last 2 decades, there is little published information to guide the selection and application of available assays. Moreover, the scarcity of fecal iron data in pediatric populations challenges the establishment of meaningful detection limits (Table 1). Acknowledging a wide age range across studies, the work by Fairweather-Tait et al. (55), Qasem et al. (57), Kalipatnapu et al. (69), and Paalanne et al. (128) provided some data related to the concentration of iron present in stool from pediatric populations. Abbasi et al. (151) emphasized the need for reliable yet feasible methods for iron quantification, underscoring the importance of comparison with advanced methods, such as comparison between ICP-MS (an automated instrument) and a commercial colorimetry kit. In addition to standardization of laboratory procedures, harmonization of the approaches used to report biomarker data is required to enhance interpretability of published findings and enable between-study comparisons (152). For example, in studies of fecal iron measurement in infants and children, there have been inconsistencies in the reported measurement units for stool iron concentrations and methods of accounting for stool consistency (e.g., mass of iron in relation to the weight of dry or wet stool) (Table 1). Assay selection may depend on the research question, study population, and logistical feasibility according to the study setting; however, validation studies that include comparison across several methods should be prioritized to address current gaps surrounding the efficiency, reproducibility, and reliability of current stool iron quantification methods. As outlined in Table 3, the general principals of each method can be readily compared in terms of general considerations (e.g., cost, feasibility, technical expertise required), but application of each method requires specific validation with respect to iron quantification in stool, including direct comparison of quality control metrics such as detection and quantification limits.

### Conclusions

Although the measurement of fecal iron can be incorporated into human microbiome research studies (including probiotic, prebiotic, or synbiotic intervention trials) as well as iron intervention trials, further research is needed to establish its usefulness in assessing the clinical and public health benefits against the risks of such interventions. Also, the choice of method to evaluate fecal iron should be driven by the required assay precision, sensitivity, and feasibility, yet such factors have been seldom reported or critically assessed to date. Among the available methods to quantify stool iron, ICP-MS is considered the most reliable, but its high cost and operational complexity requires dedicated laboratories that may be either too expensive for large-scale epidemiologic studies or inaccessible in low-resource settings where ID is a major public health problem. Use of colorimetric kits is a reliable, low-cost alternative that requires further field testing and standardization prior to its adoption as a standard method for stool iron assessment in clinical research. Stable isotope techniques remain the gold standard for measuring iron absorption and iron balance but are usually not feasible for large-scale trials. Validation and standardization of stool iron assessment methods are required to facilitate meaningful comparison across all age groups and regions. The incorporation of such analyses in future human microbiome studies may yield new insights into the interactions between intestinal microbiota and iron and thereby contribute to the development of interventions that mitigate ID and promote a healthy microbiome.

## Notes

The authors reported no funding received for this study.

Author disclosures: AZK, SB, KMO, SZ, DER. The authors have no conflicts of interest to disclose.

Abbreviations used: AAS, atomic absorption spectrophotometry; DCYTB, duodenal cytochrome B; DMT1, divalent metal transporter 1; Hb, hemoglobin; ICP-MS, inductively coupled plasma mass spectrometry; ICP-OES, inductively coupled plasma optical emission spectrometry; ID, iron deficiency; IDA, iron deficiency anemia.
